# Baseline Error Performance and Reliability of a Wheelchair Balance Task in Para Sport Athletes

**DOI:** 10.3390/healthcare14152432

**Published:** 2026-08-06

**Authors:** Ryan N. Moran, Margaret Stran, Alexandra C. Curry

**Affiliations:** Adapted Athletics, The University of Alabama, Tuscaloosa, AL 35487, USA; mstran@ua.edu (M.S.); accurry2@ua.edu (A.C.C.)

**Keywords:** sports medicine, rehabilitation, motor control, injury

## Abstract

**Background/Objectives**: Healthcare professionals have begun utilizing a wheelie task for balance assessment in para sport athletes. With recent advancements to incorporate dual-task exercises, the purpose of this study was to evaluate baseline wheelie performance during single and dual-task and measure 1-week and 45-day reliability in a sample of collegiate para sport athletes. **Methods**: Twenty-five (male n = 14, 56.0%) collegiate para sport athletes completed a wheelie assessment under single and dual-task conditions. Measures consisted of standardized biomechanical errors (e.g., front wheels touching ground, changing grip on wheel) as scored by a single clinician during eyes-open and eyes-closed positions. Assessments were repeated 7 ± 2 days and 45 ± 7 days later. **Results**: At baseline, there were no differences with dual-task exercise during eyes-open wheelie balancing (*p* = 0.096). No differences were observed from single to dual-task in the eyes-closed position (*p* = 0.154) at baseline, but there were significantly more errors during this position at both the 1-week (*p* = 0.005) and 45-day (*p* = 0.004) retest intervals. Fair to moderate correlations existed for both eyes-open and eyes-closed positions during single and dual task across 1-week (rs ≥ 0.49) and 45-day (rs ≥ 0.40) retest intervals. **Conclusions**: The incorporation of dual-task exercises during the eyes-closed wheelie position appears to increase the difficulty of balance compared to single-task, possibly making it a useful assessment to evaluate balance in para-athletes. There is fair to moderate correlation of the wheelie during single and dual tasks over time. Future research is needed on dual-task compensation post-injury.

## 1. Introduction

Balance remains a critical element of sports injury evaluation and rehabilitation, as a way for clinicians to detect and manage neurological impairment following a myriad of injuries, including sport-related concussion [1], spinal cord injury [2], stroke [3], and lower extremity neuromuscular pathologies [4]. Consensus guidelines for the evaluation of sport-related concussion focus on both static balance and dynamic gait as a measure of neurological impairment [5]. While balance and postural control tasks are routinely completed in an upright, standing position, this poses a challenge for para sport athletes given their use of a wheelchair for daily means of mobility in and out of sport.

Recent adaptations to standardized balance assessments, the Balance Error Scoring System (BESS) and its modified (mBESS) version, have been developed for wheelchair sport athletes. This Wheelchair Error Scoring System (WESS) includes a series of upright seated balance with the individual’s eyes open and closed on a firm and unstable surface [6,7]. The WESS concludes with an eyes-open and -closed wheelie task, where the individual lifts their front wheels off the ground and attempts to maintain that position for 20 s. Moran and colleagues [6] noted that the wheelie tasks of the WESS produced worse balance scores than firm-surface seating in collegiate para-athletes. Additionally, 67% of their sample committed errors during the eyes-closed wheelie, further validating its potential to challenge balance. Recent evaluation tools for sport-related concussion, the Sport Concussion Assessment Tool 6 (SCAT6) [8] and its office version (SCOAT6) [9] incorporate a dual-task exercise during gait to further disrupt balance by forcing the brain to manage both motor and cognitive tasks [8,10]. While dual-task tandem gait in collegiate athletes results in a longer time for completion and more postural sway [11,12], dual-task propulsion in a wheelchair has been noted to result in worse spatiotemporal parameters, including a shorter cycle, higher propulsion frequency, and a lower push angle, further validating the need for dual-task conditions in wheelchair motor mechanics [13].

While dual-task balancing in a wheelie has yet to be explored, the use of this condition can help to potentially detect and rehabilitate para-athletes following concussion. Since dual-task cost has been noted in more dynamic motor tasks, like tandem gait, over static balance, it is possible that dual-task wheelie balance may have similar effects over upright seated balance. Therefore, the purpose of this study was to evaluate baseline wheelie performance under single and dual tasks in a sample of collegiate para sport athletes. A secondary purpose was to establish the test–retest reliability of this novice battery at 1 week and 45 days following baseline testing. It was hypothesized that dual-task wheelie conditions would jeopardize balance and result in more balance errors than single-task. Additionally, we purported that there would be fair reliability of the wheelie across single and dual tasks over retest intervals.

## 2. Materials and Methods

### 2.1. Project Overview

This study used a cross-sectional repeated measures design of the wheelie task of the WESS. This study used a novel approach of implementing the wheelie under normal (single-task) balancing and while completing a dual-task cognitive exercise. The dual task used was “*serial integers*”, where the participants counted backwards, aloud, by 7 s, from a randomly assigned two- or three-digit number (e.g., counting backwards from 100 by 7 s = 100 − 93 − 86 − 79, etc.), for consistency with the SCAT6. If integers of 7 were too challenging and compensating balance performance, integers of 6, were then used. Integers of 5 or lower were not used to prevent any repetitious patterns or due to the potential ease of counting without task prioritization. We also aimed to establish the reliability of the wheelie during both single and dual tasks. This project was conceptualized from the prior research of Moran et al. [6] and Harper et al. [7] to further investigate the WESS and its more prominent error conditions, along with similar work that explores the incorporation of single- and dual-task mBESS performance in a collegiate athlete sample [14]. This design was determined to help clinicians understand the role that dual-task conditions may have, given the recent inclusion of dual-task balance on the SCAT6 and SCOAT6 tools for the evaluation and management of sport-related concussion [8,9]. Additionally, these findings would potentially help inform future sport-related concussion tools developed specifically for para sport athletes who use a wheelchair for daily means of mobility [15].

### 2.2. Study Recruitment and Participants

Twenty-six collegiate para-athletes aged 18 to 28 years from a single institution were recruited to participate in the study. All participants used wheelchairs as their primary means of mobility. One athlete was not able to perform a wheelie task and thus was excluded from the analyses, as a wheelie would not be practically used clinically for this athlete. Therefore, a total of 25 collegiate para-athletes (age = 21.72 ± 2.6) participated, consisting of 14 males (56.0%) and 11 females (44.0%). Although a small homogenous sample, this sample represented the entirety of the collegiate para sport program at the site. Participants were predominately from the following para sports: men’s (n = 9, 36.0%) and women’s basketball (n = 11, 44.0%), men’s track and field (n = 3, 12.0%), and men’s tennis (n = 2, 8.0%). No female para track and field or tennis athletes qualified for the study. Full demographics of the sample are provided in Table 1.

### 2.3. Data Collection and Procedures

**Figure 1:**
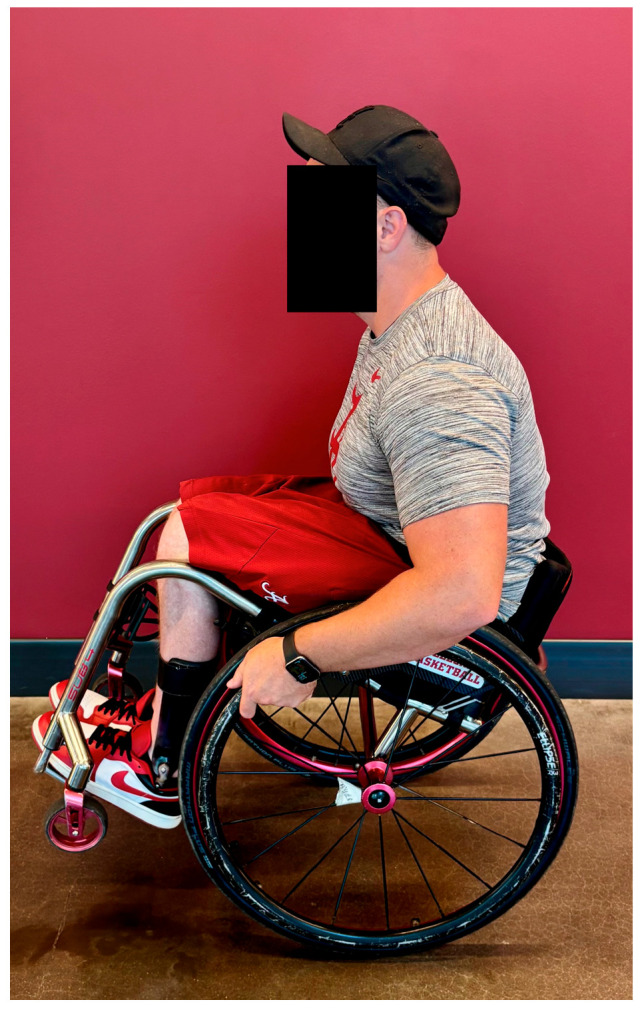
Wheelie position.

Participants arrived at the adapted athletic training facility during a designed research time communicated to the participant prior to the start of their respective sport season. Upon arriving, participants completed the informed consent, followed by providing demographics of age, sex, specific injury or pathology (e.g., spinal cord injury, spina bifida, etc.), functional classification (1.0–4.5) and concussion history. Functional classification is a rating for functional ability and trunk control, with lower classification indicating lower ability and control. Researchers then informed the participant that they would complete either the single-task or dual-task wheelie first, in a counterbalanced manner. Participants completed a wheelie (Figure 1) with their eyes open, holding it for 20 s, before returning to a grounded starting position. The wheelie was then performed again for the same time with the participant’s eyes closed. The opposite task (single or dual) was then completed after their first task, with eyes open followed by eyes closed. Balance was recorded using human-error scoring of the WESS [6], specific to the wheelie task, consisting of (i) removing hands from the wheel, (ii) sliding the hands along the wheel, (iii) coming out of the wheelie/front wheels touching the ground, (iv) remaining out of testing position for more than 5 s, and (v) opening of the eyes (eyes closed task only). If two errors were committed simultaneously, only one was counted. The number of errors per task and condition ranged from 0 (no errors) to 10, with lower scores indicating better balance. Following the initial testing day, all participants returned for retesting and performed the battery in the same order of task administration, two additional times: 7 days (1-week) ± 2 days and 45 days ± 7 days from initial testing. Retesting intervals occurred on an average of 7.08 ± 0.8 and 41.84 ± 4.1 days after initial testing. All 25 participants completed the baseline, 1-week and 45-day testing, resulting in a 100% retention rate. One scorer was used throughout all testing.

### 2.4. Data Analysis

Descriptive statistics (e.g., means, standard deviation) and confidence intervals were provided for all demographics and wheelie balance performance for ease of clinical application. Due to the non-normality of wheelie errors, nonparametric tests were used throughout. A series of Wilcoxon signed rank tests were used to compare differences in errors between conditions during single and dual-task. Spearman’s rank-order correlations were conducted to determine the strength of error scoring over initial and retest intervals, as well as Friedman tests to detect differences across initial, 1-week, and 45-day intervals. Interpretation of the correlation coefficients was ≤0.2 = weak, 0.3–0.5 = fair, 0.6–0.7 = moderate and ≥0.8 = very strong. No missing data were recorded. IBM SPSS Statistics for Mac, Version 31 (Armonk, NY, USA) was used for all statistical analyses.

## 3. Results

### 3.1. Initial Balance Error Performance

At the initial testing, there were minimal errors committed during the eyes-open condition of the wheelie (0.08 ± 0.4 errors), with only one individual committing errors, which consisted of two errors. The errors increased during the eyes-closed condition (1.16 ± 1.4 errors) and slightly under the dual-task eyes-open condition (0.28 ± 0.5). Lastly, the eyes-closed dual-task wheelie (1.48 ± 1.4 errors) had the most errors committed. The full initial testing performance is provided in Table 2.

### 3.2. Dual-Task Effects

At the initial testing, there were no differences between a single- and dual-task wheelie during both eyes-open (*Z* = −1.667, *p* = 0.096) and eyes-closed (*Z* = −1.426, *p* = 0.154) conditions (Table 3). Interestingly, there were significantly more errors committed during the dual-task eyes-closed condition at the 1-week (1.56 ± 1.4 vs. 1.04 ± 1.1; *Z* = −2.829, *p* = 0.005) and the 45-day (1.68 ± 1.2 vs. 1.00 ± 0.8; *Z* = −2.887, *p* = 0.004) retest intervals than during the single task. No dual-task differences were observed at any retest time points for eyes-open conditions (*p* > 0.05).

### 3.3. Test–Retest Reliability

There were no significant findings between error scoring across the three testing intervals for eyes-open (*p* = 0.368) and eyes-closed (*p* = 0.814) conditions under the single task. Similarly, there were no differences under the dual task for eyes-open (*p* = 0.143) and eyes-closed conditions (*p* = 0.616). Regarding the strength of each wheelie task and condition across retest intervals, there were significant fair-to-moderate correlations from the initial to 1-week (r_s_ = 0.539, *p* = 0.005) and 45-day (r_s_ = 0.524, *p* = 0.007) tests for single-task eyes-closed wheelie errors (Table 4). The strength increased to a moderate correlation (r_s_ = 0.670, *p* < 0.001) from 1-week to 45-day intervals for this same single-task eyes-closed condition. Under dual-task conditions, the eyes-closed condition strength increased from the initial to 1-week (r_s_ = 0.655, *p* < 0.001) and 45-day (r_s_ = 0.543, *p* = 0.005) tests. The single-task eyes-open condition was not considered due to only one individual making errors during this task at the initial and 1-week intervals. Under dual-task conditions, the strength was fair at the 1-week (r_s_ = 0.495, *p* = 0.012) and 45-day (r_s_ = 0.408, *p* = 0.043) intervals.

## 4. Discussion

### 4.1. Application of Baseline Findings

The initial testing interval in this study is indicative of the baseline performance, given it reduces the learning effects across repeat testing sessions, serving as a measure of normal one-time performance. Our single-task errors are similar to prior research in collegiate para-athletes, with slightly better performance in our sample. Moran et al. [6] noted 19% (n = 4/21) and 67% of collegiate para-athletes made errors on the eyes-open and eyes-closed wheelie task of the WESS, in relation to our sample with 16% (n = 1/25) and 52%, respectively. It is possible that our findings are lower, as the athletes in our study did not complete the seated out-of-wheelchair balance conditions of the WESS (e.g., seated on a firm surface and balance disk) prior to the wheelie, which may have increased the trunk fatigue in prior findings [6]; however, future research is needed to confirm this hypothesis. Harper et al. [7] published average WESS findings in sample of veteran para-athletes, noting an average score of 25 errors across the full WESS, but they did not provide specific breakdowns in errors by condition. These authors also categorized WESS performance as pass/fail based on whether 10 errors were committed in a single trial, which resulted in 64% of non-walkers and 67% of those with spinal cord injury. Again, despite no breakdown by condition, it should be noted that 0% of participants in our study met the “fail” criteria of Harper et al. [7]. It is suspected that our improved findings are based on having a younger athletic sample, as opposed to a veteran athlete sample, with an average age of 56 years, but this is hypothetical and needs further testing to examine population and age differences.

### 4.2. Innovation and Clinical Implementation

This project examined the implementation of a dual-task wheelie condition of the WESS for exploratory baseline purposes in clinical injury evaluation and rehabilitation. Similar research around postural control using the mBESS in collegiate athletes (non-para-athletes) noted no dual-task effects on errors or postural sway during double leg, single leg, and tandem stance balancing [14], hinting that dual-task conditions may be more sensitive to dynamic motor control tasks, such as walking tandem gait [12,16], as opposed to upright balancing. It is plausible that the wheelie task further challenges balance over seated upright postural control on the WESS, given Moran et al. [6] noting the wheelie condition elicited the most errors and a higher prevalence of errors committed throughout the WESS. Interestingly, there were no differences from single to dual task with eyes open or closed at initial testing, which would mirror the prior findings in more static balance assessments. However, dual-task differences were observed during the eyes-closed conditions at both the 1-week and 45-day retest, indicating that a dual-task cognitive exercise during an eyes-closed wheelie may further challenge the central nervous system and somatosensory systems of the body. A hypothesis for the lack of baseline differences may be attributed to a learning effect or the potential for the baseline performance to be near its ceiling for the sample. The sample’s eyes-closed single-task mean scores decreased over each subsequent retest interval, while the dual-task scores increased. We hypothesize that a lack of dual-task differences at initial testing with apparent differences later may be due to fatigue accumulation or reduced cognitive resources over time make dual-tasking harder and thus more errors [17]; however, these were not investigated in the current study. It is possible that compensatory strategies may have also played a role, such as prioritizing balance first and prioritizing the cognitive exercise more at retesting, which would produce more errors, but future research is needed to confirm this hypothesis. Additionally, the dual-task cost may become more prominent as single-task balancing improved, creating a disparity between the two principles. Regardless, this is important, as dual-task differences do not appear to diminish early on and thus can be used as an effective way to challenge balance and motor control in para-athletes following neurological injury over time.

The final aim of this study was to examine the reliability of the wheelie task under single- and dual-task conditions. With the wheelie task being the WESS item that elicited the most errors in prior research [6], single-task data can provide a further context to prior WESS findings. Naturally, there is a slight decline in the strength of the error performance from initial to 1-week and 45-day retesting. A mixture of increases and decreases in strength was present from 1 week to 45 days, indicating a fluctuation in balance over time and possibly variances by daily factors such as fatigue, sleep, or soreness. This is a potential contributor towards the decreased sensitivity of the mBESS at 3 days post-concussion [18]. Our findings are similar to 1-week reliability data in the mBESS, with fair to moderate correlations in single-leg and tandem stance during both single- and dual-task conditions [14]. Various other wheelchair skills tests have demonstrated fair to good reliability as well, including the Wheelchair Skills Test [19], Adapted Manual Wheelchair Circuit [20], Limits of Stability and sequential weight shifting in a wheelchair [21], along with a self-reported ability to perform a wheelie over a 1-month period [22].

Balance remains a critical element of injury evaluation and rehabilitation in all athletes, regardless of the disability status, sex, age, or other factors. The findings of this study enable clinicians to incorporate the use of dual-task exercises with clinical examinations and management in para-athletes. Given that standardized tools, such as the balance examination on the SCAT6 and SCOAT6, are not able to be administered in wheelchair-using para-athletes, these findings help provide more evidence into the use of the WESS to supplement the mBESS and tandem gait [23,24]. Additionally, as tandem gait in its single and dual tasks is also not able to be administered to this sample, the dual-task wheelie may be able to provide clinically useful evidence to support its use, as a supplement to dual-task tandem gait in regard to the management of sport-related concussion and musculoskeletal injury.

### 4.3. Limitations and Future Research

This study was not without limitations. First, the results of this study are only applicable to university para-athletes in the sports of wheelchair basketball, tennis, and track and field. Different levels of competition, such as adolescent or paralympic sports, may have varying levels of balance. Second, we did not consider the amount of time (e.g., months, years) that the participants had been using a wheelchair and their experience and comfort performing wheelies. Additionally, the potential influence of cognitive task difficulty on balance outcomes was not controlled for, and our methodology only used serial integers, as opposed to other cognitive tasks widely used in concussion evaluation, such as words spelled backwards or months recited backwards. We also did not count cognitive errors, which limits the understanding of the dual-task cost and prioritization of cognition or balancing. Lastly, additional factors such as the absence of assessor blinding, a single-clinician scoring approach, and lack of objective biomechanical validation, may have impacted the results. These results are only able to be applied to baseline assessments, as middle and post-season balance performance may differ due to fatigue or overuse injuries. Future research should consider the effects of single- and dual-task wheelies performed following concussion or neurological injury to help understand post-injury balance during the acute and sub-acute phases of injury and recovery time points (e.g., return-to-play). As the wheelie can be used for comprehensive rehabilitation, it is important for future studies to address potential modifiers of performance, such as body mass index, prior neurological injuries, core and upper extremity strength, etc., that may alter how para-athletes perform prior to and following injury. Lastly, future studies should aim to incorporate objective biomechanical measurements using instrumented pressure mapping or force plate technology, along with the development of normative values across sport, sex, classification, and disability/pathology diagnoses.

## 5. Conclusions

In conclusion, the incorporation of a dual-task cognitive exercise during a wheelie where the patient has their eyes closed further increased the difficulty of balance and motor control, compared to a single task. This provides a baseline context for a wheelie under varying conditions to potentially better detect balance impairments to be used in injury evaluation and rehabilitation. There is fair to moderate correlation of the wheelie during single- and dual-task conditions that provides baseline reliability over time. Future research is needed on dual-task compensation during the acute and sub-acute phase of recent injury, as our findings only consist of exploratory baseline data.

## Figures and Tables

**Table 1:** Demographics of the sample (n = 25).

Characteristic	N (%)
Sport	-
Wheelchair basketball	20 (80.0)
Wheelchair tennis	2 (8.0)
Wheelchair track and field	3 (12.0)
Concussion history	-
* *No prior history	19 (76.0)
* *1 prior concussion	3 (12.0)
* *2+ prior concussions	3 (12.0)
Functional classification	-
* *1.0	7 (28.0)
* *1.5	3 (12.0)
* *2.0	3 (12.0)
* *2.5	3 (12.0)
* *3.0	5 (20.0)
3.5	2 (8.0)
4.0	1 (4.0)
4.5	1 (4.0)
Pathology	-
* *Spinal cord injury	12 (48.0)
* *Spina bifida	6 (24.0)
* *Transverse myelitis	2 (8.0)
* *Other	5 (20.0)

**Table 2:** Initial wheelie error performance for the sample.

Task	Condition	% of Participants with Errors Committed				
		0	1	2	3	4+
Single	Eyes-Open	96.0	-	4.0	-	-
	Eyes-Closed	48.0	24.0	4.0	12.0	12.0
Dual	Eyes-Open	76.0	20.0	4.0	-	-
	Eyes-Closed	20.0	44.0	20.0	8.0	8.0

**Table 3:** Error performance and dual-task differences across time intervals.

Time Interval	Task	Conditions	Mean ± SD	95% CI	Median (IQR)	*p*
Initial	Single	Eyes-Open	0.08 ± 0.4	0.00, 0.25	0 (0)	-
		Eyes-Closed	1.16 ± 1.4	0.56, 1.76	1 (3)	
	Dual	Eyes-Open	0.28 ± 0.5	0.06, 0.50	0 (1)	0.096
		Eyes-Closed	1.48 ± 1.4	0.91, 2.05	1 (1)	0.154
Day 7	Single	Eyes-Open	0.08 ± 0.4	0.00, 0.25	0 (0)	-
		Eyes-Closed	1.04 ± 1.1	0.57, 1.51	1 (2)	
	Dual	Eyes-Open	0.08 ± 0.2	0.00, 0.19	0 (0)	1.000
		Eyes-Closed	1.56 ± 1.4	0.98, 2.14	1 (1)	0.005
Day 45	Single	Eyes-Open	0.12 ± 0.4	0.00, 0.30	0 (0)	-
		Eyes-Closed	1.00 ± 0.8	0.64, 1.36	1 (1)	
	Dual	Eyes-Open	0.24 ± 0.5	0.02, 0.46	0 (0)	0.180
		Eyes-Closed	1.68 ± 1.2	1.18, 2.18	2 (1)	0.004

Note: *p*-value is for differences between single- and dual-task conditions.

**Table 4:** Correlation strength of errors across time intervals.

	Task	Conditions	Day 7	Day 45
Initial	Single	Eyes-Open	1.000	0.722
		Eyes-Closed	0.539	0.524
	Dual	Eyes-Open	0.495	0.408
		Eyes-Closed	0.655	0.543
Day 7	Single	Eyes-Open	-	0.722
		Eyes-Closed		0.670
	Dual	Eyes-Open		0.558
		Eyes-Closed		0.426

Note: All items, besides the initial eyes-open single-task condition, were significant at *p* ≤ 0.05.

## Data Availability

De-identified data from this study are not available for public access. Data availability inquiries are subject to institutional review board and legal compliance approval, and requests can be emailed to the corresponding author.

## Abbreviations

The following abbreviations are used in this manuscript:

mBESS	Modified Balance Error Scoring System
SCAT6	Sport Concussion Assessment Tool 6
SCOAT6	Sport Concussion Office Assessment Tool 6
WESS	Wheelchair Error Scoring System
